# Supplementary material for: Bacterial second messenger 3′,5′-cyclic diguanylate attracts Caenorhabditis elegans and suppresses its immunity
Source: Commun Biol. 2020 Nov 20;3:700. doi: 10.1038/s42003-020-01436-9 (PMC7679379; doi:10.1038/s42003-020-01436-9)

Supplementary Figure 1. C-di-GMP does not affect the growth of *E. coli* OP50.

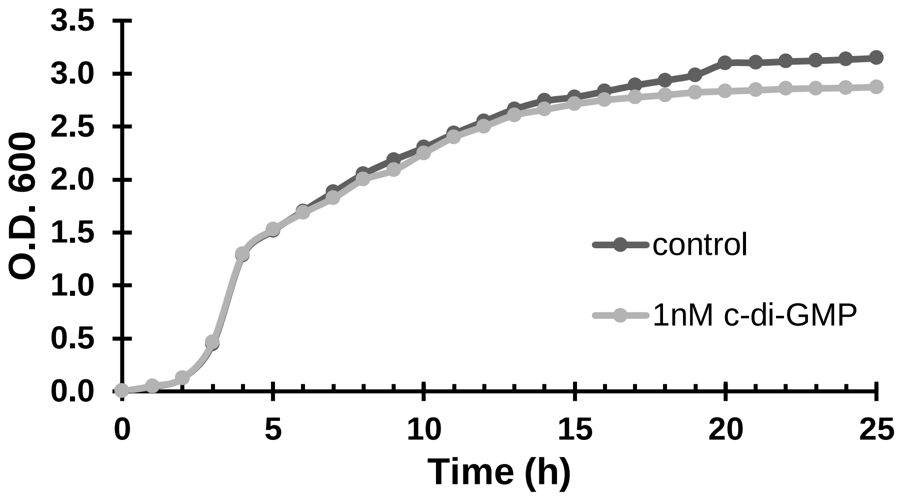

Supplementary Figure 2. C-di-GMP does not enhance the surface attachment of *E. coli* OP50.

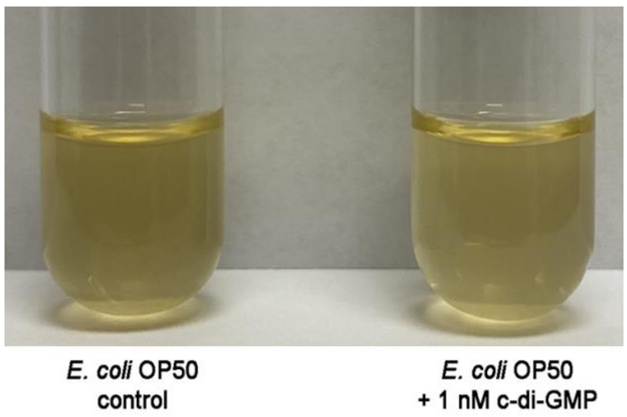

Supplementary Figure 3. Lifespan of N2 worms fed on untreated *E. coli* OP50 and treated *E. coli* OP50 that was grown overnight with 1 nM c-di-GMP.

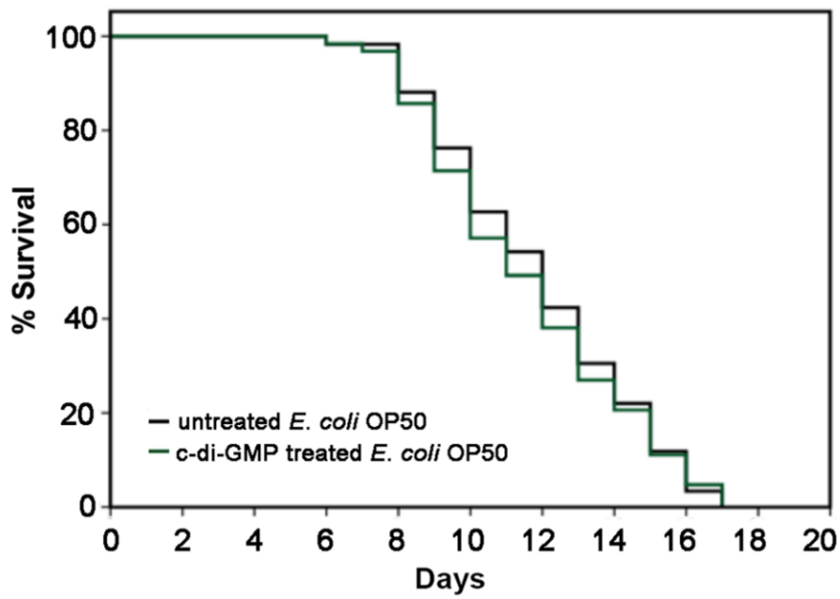

Supplement: Supplementary file 1 — Supplementary Information [file 42003_2020_1436_MOESM1_ESM.pdf]
