# Supplementary material for: Bacterial second messenger 3′,5′-cyclic diguanylate attracts Caenorhabditis elegans and suppresses its immunity
Source: Commun Biol. 2020 Nov 20;3:700. doi: 10.1038/s42003-020-01436-9 (PMC7679379; doi:10.1038/s42003-020-01436-9)
Supplement: Supplementary file 3 — Description of Additional Supplementary Files [file 42003_2020_1436_MOESM3_ESM.pdf]

Description of Additional Supplementary Files

File Name: Supplementary Data
Description: Contains source data underlying the graphs in figures.
