# Supplementary material for: Bacterial second messenger 3′,5′-cyclic diguanylate attracts Caenorhabditis elegans and suppresses its immunity
Source: Commun Biol. 2020 Nov 20;3:700. doi: 10.1038/s42003-020-01436-9 (PMC7679379; doi:10.1038/s42003-020-01436-9)
Supplement: Supplementary file 5 — Reporting Summary [file 42003_2020_1436_MOESM5_ESM.pdf]

natureresearch

Corresponding author(s): DBPR COMMSBIO-20-0477A

Last updated by author(s): 10/07/2020

Reporting Summary

Nature Research wishes to improve the reproducibility of the work that we publish. This form provides structure for consistency and transparency in reporting. For further information on Nature Research policies, see Authors & Referees and the Editorial Policy Checklist .

Statistics

For all statistical analyses, confirm that the following items are present in the figure legend, table legend, main text, or Methods section.

n/a	Confirmed	
☐	☒	The exact sample size (*n*) for each experimental group/condition, given as a discrete number and unit of measurement
☐	☒	A statement on whether measurements were taken from distinct samples or whether the same sample was measured repeatedly
☐	☒	The statistical test(s) used AND whether they are one- or two-sided<br>*Only common tests should be described solely by name; describe more complex techniques in the Methods section.*
☐	☒	A description of all covariates tested
☒	☐	A description of any assumptions or corrections, such as tests of normality and adjustment for multiple comparisons
☐	☒	A full description of the statistical parameters including central tendency (e.g. means) or other basic estimates (e.g. regression coefficient) AND variation (e.g. standard deviation) or associated estimates of uncertainty (e.g. confidence intervals)
☐	☒	For null hypothesis testing, the test statistic (e.g. *F*, *t*, *r*) with confidence intervals, effect sizes, degrees of freedom and *P* value noted<br>*Give P values as exact values whenever suitable.*
☒	☐	For Bayesian analysis, information on the choice of priors and Markov chain Monte Carlo settings
☒	☐	For hierarchical and complex designs, identification of the appropriate level for tests and full reporting of outcomes
☒	☐	Estimates of effect sizes (e.g. Cohen's *d*, Pearson's *r*), indicating how they were calculated

Our web collection on statistics for biologists contains articles on many of the points above.

Software and code

Policy information about availability of computer code

Data collection	No software was used.
Data analysis	Kaplan Meier Survival analysis conducted on SPSS 21.0.0.0 64-bit.<br>For choice index assays, unpaired student's t-test (between two groups) and one-way balanced ANOVA (for more than two groups) were used for statistical analysis in Microsoft Excel. p-value < 0.05 was accepted as statistically significant.<br>For gut colonization and qRT-PCR, the data were pooled and analyzed in Microsoft Excel using unpaired Student's t-test (between each target gene and the control act-1), and a p-value < 0.05 was accepted as statistically significant.

For manuscripts utilizing custom algorithms or software that are central to the research but not yet described in published literature, software must be made available to editors/reviewers. We strongly encourage code deposition in a community repository (e.g. GitHub). See the Nature Research guidelines for submitting code & software for further information.

Data

Policy information about availability of data

All manuscripts must include a data availability statement. This statement should provide the following information, where applicable:

- Accession codes, unique identifiers, or web links for publicly available datasets
- A list of figures that have associated raw data
- A description of any restrictions on data availability

The authors declare that all data supporting the findings of this study are available within this article, Supplementary material, and Supplementary data set. Source data underlying the graphs in figures are provided in Supplementary data set.

Field-specific reporting

Please select the one below that is the best fit for your research. If you are not sure, read the appropriate sections before making your selection.

- [x] Life sciences
- [ ] Behavioural & social sciences
- [ ] Ecological, evolutionary & environmental sciences

For a reference copy of the document with all sections, see nature.com/documents/nr-reporting-summary-flat.pdf

Life sciences study design

All studies must disclose on these points even when the disclosure is negative.

Sample size	Lifespan assays were conducted with samples sizes of 180-200 L4 young adult worms with around 60 worms per replicate (Lorenzo Galluzzi et al. (eds.), Cell Senescence: Methods and Protocols, Methods in Molecular Biology, vol. 965, DOI 10.1007/978-1-62703-239-1_31). This ensured a representative population that would yield statistically reliable survival data.<br>Sample sizes for choice index assays were determined based on previous work. Sample sizes ranged based on synchronization and how many eggs were laid, but this usually fell somewhere between 50-100 L4 worms, where populations of young adults were split into appropriate numbers so similar size populations were present on each plate in triplicate. Sample size ensured reliable statistical analysis. (Zhang X, Zhang Y. DBL-1, a TGF-beta, is essential for caenorhabditis elegans aversive olfactory learning. Proc Natl Acad Sci U S A 2012 Oct 16;109 (42):17081-6).<br>Sample sizes for differential gene expression analysis was around 300-400 L4 young adults to ensure appropriate quality and quantity of RNA to construct cDNA.
Data exclusions	No data were excluded in this study.
Replication	All experiments described in this study were repeated at least three times
Randomization	C. elegans were selected entirely at random from a synchronized population prior to any experimental procedures.
Blinding	All experiments were single-blind assays.

Reporting for specific materials, systems and methods

We require information from authors about some types of materials, experimental systems and methods used in many studies. Here, indicate whether each material, system or method listed is relevant to your study. If you are not sure if a list item applies to your research, read the appropriate section before selecting a response.

Materials & experimental systems

n/a	Involved in the study
x	Antibodies
x	Eukaryotic cell lines
x	Palaeontology
	x Animals and other organisms
x	Human research participants
x	Clinical data

Methods

n/a	Involved in the study
x	ChIP-seq
x	Flow cytometry
x	MRI-based neuroimaging

Animals and other organisms

Policy information about studies involving animals; ARRIVE guidelines recommended for reporting animal research

Laboratory animals	The only animal used in the study is the invertebrate nematode, C. elegans, a commonly-used lab model.
Wild animals	The study did not involve wild animals.
Field-collected samples	The study did not involve samples collected from the field.
Ethics oversight	No ethical approval was required for working with C. elegans.<br>Clemson University Institutional Biosafety Committee approved the protocol of using BSL-2 pathogens to feed C. elegans.

Note that full information on the approval of the study protocol must also be provided in the manuscript.
